# Some of the Common Forms of Headache1Read before the Rochester Pathological Society, October 6, 1904.

**Published:** 1904-11

**Authors:** A. B. Frazee

**Affiliations:** Rochester, N. Y., Formerly Assistant in the Philadelphia Polyclinic, Eye Department.


### Some of the Common Forms of Headache.[1]

By A. B. FRAZEE, M. D., Rochester, N. Y.,
Formerly Assistant in the Philadelphia Polyclinic, Eye Department.

IT IS a frequent experience with all physicians in general practice to be called upon by patients wishing to be relieved of headache, who, aside from this one ailment, believe themselves to be in good health. Still more often does the pharmacist meet with the demand for the headache powders, migraine tablets, bromo-seltzers and the like, which are extensively advertised and sold for the cure of this complaint. Many of these sufferers have tried one physician after another without avail, and at last have resorted to the patent medicines, which, at least for the time being, relieve their distress. That this is unscientific and injurious to health, is well known by the profession; but the patients, rendered desperate by their sufferings, are willing to take risks in order to secure present relief. In every community a vast amount of misery is caused by headache alone, and every practitioner has among his patients those who are more or less unfitted for work and the enjoyment of life on this account.

The child in school sometimes suffers so constantly that he cannot pay the necessary attention to his studies, and so is rated as dull and backward. He falls behind those of his age, becomes discouraged, and, as soon as possible, gives up school altogether, sacrificing what otherwise might have been a successful career because of the headaches which render him unfit for study. The bookkeeper, or business man may be obliged to give up a lucrative position because of the headaches induced by his work; or the mother may be, herself, so miserable as to exert a depressing effect on the whole household. Fortunately, such catastrophies are not so common now as formerly, this being due to the fact that more accurate diagnoses are being made, and more rational treatment instituted. The subject is, therefore, well worth our attention, and a patient coming to us with this complaint should not be lightly dismissed.

Of course, it is admitted that headache is merely a symptom, and that its successful treatment depends upon our detecting the cause, and removing or neutralising it as fully as possible. But as it is the predominant symptom in a variety of pathological states, and is frequently the only thing which drives the patient to seek relief, it is worthy of consideration and study by itself.

Authorities are not fully agreed as to the classification of headaches, but it seems to me that the following will cover most, if not all cases: (1) toxic headaches, met with in the course of

---

1. Read before the Rochester Pathological Society, October 6, 1904.

feverish complaints, of uremia, constipation and the like; this would also include the headaches of gout and rheumatism; (2) headaches occasioned by disturbances of blood pressure in the brain, causing cerebral congestion or anemia; (3) headaches produced by irritations or diseases of the organs of special sense—eye, nose, and ear; (4) neurotic headaches from hysteria, neurasthenia and allied disorders; (5) headaches produced by brain diseases and lesions, such as tumors, meningitis, syphilis and the like.

As the subject is far too extensive to be contained within the limits of a single paper, I shall take the liberty of choosing a few of the common varieties which in many respects resemble one another, and shall consider them mainly from the point of diagnosis and, incidentally, of treatment.

Probably the most common of all headaches are those which I have classified as toxic in origin. There is scarcely a disease, attended by fever, that is not also accompanied by headache at some time in its course. Most of these are so clearly dependent on the original malady that the cause is recognised even by the patient himself; but at times the disease is so masked that for a long time it goes undetected. This is often the case in uremia, in indigestion and constipation, and these possible causative conditions should always be borne in mind whenever a persistent headache is presented for treatment. The character of the pain has very little diagnostic significance. It may be dull and diffused, or sharp and localised. An item of greater significance is the fact that it is apt to occur early in the morning, thus distinguishing it to a certain extent from cerebral congestion or eye strain, both of which are apt to come on a few hours after rising, or in the latter half of the day. But a positive diagnosis can only be arrived at by making a urinary examination, or by relieving the constipation and indigestion and noting the effect on the head.

Of the headaches dependent upon altered blood pressure within the brain, I believe the hyperemic form to be very common. The diagnosis of congestive headache is often very easy, it being characterised by flushed face, shining eyes, a feeling of pressure about the head, and often subjective sensations of ringing or roaring in the ears and spots before the eyes. Many of these patients are subject to nose bleed, and tell us that the loss of blood relieves the pain for the time being. The heat of the sun, or a stooping posture increases the distress, and the sufferer likes to sleep with his head high. Cold applications to the forehead are soothing, while hot cloths are apt to increase the distress. These are the symptoms of a typical case, but there are occasionally patients suffering from congestion who present many of the symptoms of anemia. One instance especially comes to mind, of a spare, sallow woman, who lacked the tinnitus and throbbing above described, whose case I thought to be that of anemia, and prescribed nitroglycerin. The first dose was the last, for the pain was so suddenly and alarmingly increased that the patient and her attendant were severely frightened, and came very near telling me that my attentions were no longer required. The remedy was discontinued, and the bromides in full doses substituted, with prompt relief, thus making a diagnosis through the effect of the drug.

Occupation is an important factor in the causation of these headaches, and in the diagnosis, they being most frequently met with in people whose work produces an increased flow of blood to the brain. This is one of the varieties most frequent in business men, professional men, and students. This is the preacher's headache, and it is noticeable that the pain is sometimes present upon rising Monday morning, thus resembling the toxic headache in this respect. This is also the headache of the laborer, who scarcely thinks at all; but whose work in the hot sun sometimes results in cerebral congestion. Remedies for this variety are quite satisfactory, the list beginning with the bromides, alone or combined with ergot and the local application of cold. Doubtless, the old-fashioned remedy of bleeding would be often satisfactory, and it is not very uncommon to have some old person suggest this as having relieved him in earlier years. It is worthy of note that the coal tar antipyretics will often relieve this variety, the so-called migraine tablets being quite effectual, especially when combined with the bromides. While I believe their employment is unscientific, the effect being to mask the pain without removing the cause, yet the occasional employment of phenacetin or acetanilid will do no harm, and will afford a measure of relief while the more rational remedies are taking effect.

It would seem at first that cerebral anemia, being the opposite condition from that just described, would be easy to distinguish from hyperemia; but this is not always the case. There may be similar sensations of flashes of light, roarings in the ears, dizziness, nausea and confusion of ideas, to those found in congestion, and we can only arrive at a correct diagnosis by considering the history of the patient, his general appearance, and perhaps making a microscopical study of his blood.

Another class of headaches, namely—those produced by irritations or diseases of the organs of special sense, also includes a multitude of sufferers as its victims. I shall pass over those caused by diseases of the nose, throat and ears, and consider those caused by the eye; more especially the headache of eye strain.

Only within comparatively recent times has eye strain been recognised as a frequent cause of headache; and even now this is often overlooked and the patient goes on unrelieved for years, perhaps at last to be told by some neighbor, who has had like experience, that the real source of his suffering is in his eyes. The writer recalls a monograph, published not many years ago, describing several dozens of varieties of headache, and elaborating on the treatment, but which never mentioned, from one cover to the other, that it was ever caused by eye strain. Fortunately, the profession and the public are much more quick to recognise the affection at the present day.

To make a diagnosis of ocular headache does not, as a rule, demand special training in diseases of the eye; but it is necessary that we bear in mind the mode in which this affection is produced: otherwise, some of the most important diagnostic points will probably be overlooked, or, if noted, their true significance will be imperfectly understood.

How is this form of headache produced? A general answer to this question will be that it is caused by excessive and prolonged action of some of the muscles of the eye, and mostly in three classes of cases:

1. Where the refraction of the eye is irregular, so that a distorted image is thrown on the retina; especially where this astigmatism is of moderate degree and capable of being corrected wholly, or in part, by action of the ciliary muscle.

2. Where the refraction of the eye is not perfectly adapted to the length of the eyeball, the latter being generally a little too short, as in far-sighted people. In this case, if the disproportion is not too great, the ciliary muscle is able, by a little extra tension, to produce a perfect image on the retina.

In both of these instances it should be noted that slight or moderate astigmatism or hyperopia, capable of correction by ciliary contraction, is most liable to produce headache; while defects too great to be corrected by this means are not nearly so liable to cause pain. It should also be remembered that in these cases an extra effort on the part of the ciliary muscle is often able to give perfect, or at any rate fairly satisfactory vision, for all objects both near and far.

3. Where the motor muscles of the eyeball are unevenly balanced, so that one set has to be abnormally tense in order to keep the eyes in proper focus. In this, as in the preceding cases, it should be remembered that moderate inequalities, capable of correction by voluntary muscular effort, are the ones that result in headache. When the tendency to squint is so great that the patient is no longer able to overcome it, the effort of the muscles to straighten the eyes is abandoned, and undue strain abolished. It is, therefore, the latent squints, wholly correctable by muscular effort, that result in sensory disturbances. In these cases the pain is not always referred to the muscles involved, but is mostly reflex in character, and felt in regions more or less remote.

*Symptoms.*—Headache due to eye strain is not of the same character in all cases. Generally it is frontal, being located just over the brows, or back of the orbits, and is about the same in intensity on both sides of the head. In a smaller number the pain is in the temples, and in a still smaller percentage it is vertical, occipital, or even referred to the back of the neck. Frequently there is nausea, and rarely vomiting, thus simulating an attack of so-called migraine. The pain may be acute, but is often dull and uniform, with a sense of pressure, but lacking the tense and throbbing feeling with tinnitus, characteristic of cerebral hyperemia. There is frequently a feeling of soreness and pain in the eyeballs, and sometimes dizziness, though this is by no means constant.

It will be seen that the above described symptoms present little that is characteristic; and as they comprise about all that the ordinary patient thinks worthy of mention, it might seem impossible to arrive at a correct diagnosis. But there are a few important points which the examiner should bear in mind, the consideration of which will materially aid him in coming to a correct conclusion.

First in importance, perhaps, is the occupation of the patient, and this will be readily understood when the true nature, or modus operandi, of this form of headache is borne in mind. It has been said that excessive muscular action, either of the focusing muscles of the lens, or the motor muscles of the eye, is generally the cause of the pain. We should, therefore, expect to find headache among those persons who require to see objects distinctly, for long periods, especially at short distances. Typical of this class is the student, whose daily task it is to pore over the small letters and characters in his books, and it is a common experience to learn that his headache commenced with the beginning of school life. This affection in a student should therefore at once cause us to suspect an ocular origin, and to investigate the matter thoroughly if his trouble does not promptly yield to medical treatment.

But it is not always that headache begins during the first term in school. The high accommodative power of the child often enables him to stand the muscular strain for a number of years, and he only begins to suffer from it at about the age of puberty, or even some years later. Frequently, school life is passed in comparative comfort, and serious symptoms do not show themselves until business life begins. Especially do we look for headache among bookkeepers, stenographers and all who use their eyes for prolonged periods on near objects, almost the same class of persons as those who are subject to congestive headaches. These patients, upon being questioned, will generally admit that their distress is produced or aggravated by near work, but they seldom realise its diagnostic value, and often fail to mention it unless directly questioned on this point.

Headache, occurring soon after an attack of exhausting disease, should turn our thoughts to the eyes as a cause, especially if, on questioning, we find that the period of convalescence was beguiled by reading or fancy work. I recall many instances, especially among adults, where the eye symptoms first showed themselves after an attack of grip. The reason for this is easily explained, for the eye muscles, like the rest of the muscular system, are weakened by the disease, and if put to work too soon are liable to become seriously impaired. It is, therefore, a safe rule for convalescents from exhausting diseases, to give their eyes as long a rest as the other parts of the muscular system. Unfortunately, the rule is often broken, with resulting permanent impairment of ocular muscles, and consequent headache.

Another symptom, seldom mentioned by the patient, but easily elicited by questioning, is the frequent blurring, or clouding of the sight during prolonged use of the eyes. The patient sees clearly for a time, but the weakened muscles cannot long endure the undue tension, and relax from time to time, permitting the retinal image to become indistinct. Frequently the sufferer will look at a distance for a moment, and then be able to resume his reading. Or he may experience a sense of relief by rubbing or pressing the eyeballs. Blurring from near work is such a frequent and characteristic symptom that it should always be inquired into.

We must not be led astray by the statements which these patients often make, that they can see perfectly objects both near at hand and at a distance. This statement may be correct, and, indeed, some of these sufferers have exceptionally accurate sight. When the test card is placed before them they read the letters perhaps a line or two below those which can be distinguished by the average normal eye, and triumphantly adduce this as proof that their eyes are all right, and the headache is from other causes. And yet they may be getting this perfect vision at the expense of a constant accommodative strain, which is never relaxed during their waking hours, and which keeps up just enough local irritation to produce a feeling of weariness, heaviness or actual pain. As has been mentioned before, it is these sharp sighted people who are more prone to eye strain than those whose sight is so poor that accommodative effort will not produce a great amount of betterment, and who therefore are content with what they can see easily, and are not constantly straining for the unattainable.

Sometimes a patient with symptoms of chorea, or other nervous derangement, or who is pale and strumous and a sufferer from headache, is dosed for months, while the medical attendant entirely overlooks the fact that all these nervous symptoms are the result of eye strain, and can be removed by correcting the underlying condition.

It will be noticed that little has been said about the use of test type and test glasses to arrive at a diagnosis of strain, although they will be the final resort to confirm or disprove the diagnosis when made, for, without the aid of a mydriatic to paralyse the accommodation, the test is apt to be inconclusive, and it has been the design of this paper to describe the symptoms by which any practitioner, unequipped with special apparatus can recognise the affection with a considerable degree of confidence.

Finally, we quite often encounter persons who are suffering from two kinds of headache, the two being manifested at the same time or alternately. Especially are we apt to find the combined symptoms of eye strain and congestion, and then we must not only correct the ocular trouble, but combine with this a course of bromides to correct the hyperemic tendency. Either plan of treatment, if pursued alone, would result in disappointment.

I have omitted mention of migraine as one of the frequent forms of headache, for the reason that, as our diagnostic skill becomes greater, the number of cases of true migraine, (provided that there are any such cases), becomes less. In reading even recent articles on migraine one is impressed by the close similarity of the symptoms to many of our cases of eye strain, and it is probable that the term will eventually be dropped from the list of diseases.

698 St. Paul Street.

---

### LACERATIONS OF THE CERVIX AND THEIR CONSEQUENCES.

Dr. John W. Taylor, (*British Gynecological Journal,* November, 1903,) remarks that although the subject belongs rather to minor gynecology, it has an important bearing on what is, perhaps, still the gravest disease to which a woman is subject—puerperal septicemia—and that, in this way laceration of the uterine cervix becomes a not infrequent cause of death.

